# Autonomous slip control inspired by human physiology for improved shared control strategy

**DOI:** 10.1017/wtc.2025.10007

**Published:** 2025-06-09

**Authors:** Joana Matos, Patricia Capsi-Morales, Cristina Piazza

**Affiliations:** 1 Faculty of Engineering, University of Porto, Porto, Portugal; 2 Department of Computer Engineering, School of Computation, Information and Technology, Technical University of Munich, Munich, Germany; 3 Munich Institute of Robotics and Machine Intelligence, Technical University of Munich, Munich, Germany

**Keywords:** grasping safety, tactile sensors, prostheses, slip detection, shared autonomy control

## Abstract

The human hand is an intricate anatomical structure essential for daily activities, yet replicating its full functionality in upper-limb prostheses remains a significant challenge. Despite advances in mechanical design leading to more sophisticated and dexterous artificial hands, difficulties persist in effectively controlling these prostheses due to the limitations posed by the muscle conditions of their users. These constraints result in a limited number of control inputs and a lack of sensory feedback. To address these issues, various semi-autonomous control strategies have been proposed, which integrate sensing technologies to complement traditional myoelectric control. Inspired by human grasping physiology, we propose a shared control strategy that divides grasp control into two levels: a high-level controller, operated by the user to initiate the grasp action, and a low-level controller, which ensures stability throughout the task. This work focuses specifically on slip detection methods, introducing improvements to the low-level controller to enable more autonomous grasping behavior during object holding. The proposed slip module uses distributed 3D force sensors across the artificial hand and integrates a friction cone strategy to ensure an appropriate shear-to-normal force ratio with bandpass filtering for establishing an initial stable grasp model without prior knowledge. Experimental evaluations consist of the comparison of this novel controller with conventional state-of-the-art approaches. Results demonstrate its efficacy in preventing slippage while requiring less grasping force than previous methods. Additionally, a qualitative validation was conducted to assess its responsiveness compared to human grasping reactions to unexpected weight changes, yielding positive outcomes.

## 1. Introduction

Each year, over 20,000 individuals in the USA and EU undergo upper limb amputation (Raspopovic et al., 2021), a life-altering event that significantly impacts their autonomy, hindering their ability to engage in social, occupational, and daily activities. Despite upper limb prostheses being commonly used to mitigate these limitations, the most sophisticated devices still exhibit substantial deficiencies in functionality compared to biological hands (Cordella et al., 2016). While recent technological advancements have enabled prostheses to achieve some level of dexterity akin to the human hand (Capsi-Morales et al., 2023), the primary limitation lies in the human–prosthesis interfacing (Mendez et al., 2021). Commercial prostheses typically rely on only one or two surface-mounted electromyography (EMG) electrodes placed on the forearm for control. This reduced number of inputs results in the use of complex and nonintuitive control methods, such as muscle co-contraction and contraction speed, to operate multi-fingered hands with various available grasping patterns (Atzori and Müller, 2015).

Restoring functionality also faces significant challenges due to the disruption of biological efferent and afferent pathways, responsible for closing the loop between motor control and sensory feedback. Due to insufficient tactile information, prosthesis users often rely on visual and auditory cues to estimate grip force (Wijk and Carlsson, 2015). Research has explored closed-loop controllers that gather tactile data from the prosthesis and convey part of it to the user through vibrational motors or other modalities that act on the peripheral area. Providing haptic feedback has demonstrated certain benefits (Kim and Colgate, 2012; Clemente et al., 2015), particularly in enhancing the sense of ownership of artificial limbs. However, the time required for delivering feedback, processing biosignals, and actuation delays results in a reaction time of about 1 s, which exceeds the human physiological limits. Additionally, this approach is usually associated with increased cognitive load (Seminara et al., 2023).

Recently, strategies integrating artificial intelligence are being developed to achieve more intuitive control of sophisticated devices, leveraging their full mechanical capabilities to closely resemble biological human hands. One approach involves implementing shared control strategies, which distribute the “intelligence” of the prosthesis between the user and the robot (Cipriani et al., 2008). This transfer of decision-making and high-level understanding from the user to the prosthetic device enhances dexterity and resilience (Mendez et al., 2021). By integrating information about the hand configuration and forces of interaction, automated control processes aim to reduce the cognitive burden experienced by individuals with limb loss (Thomas et al., 2023). The estimation of grip force has been applied across various modalities, including the use of tactile sensors to optimize object contact (Kappassov et al., 2015; Sommer and Billard, 2016), analysis of hand posture (Della Santina et al., 2017), interpretation of audio signals (Zöller et al., 2018), or the implementation of cognitive vision systems for automatic selection of grasp types and aperture sizes (Došen et al., 2010; Weiner et al., 2022).

A common application of shared control approaches is known as tactile slippage control, used to ensure grasp stability. Inspired by the human nervous system and decision-making process, these methods typically involve multiple control levels pertinent to the grasping phase (Gentile et al., 2022), with a low-level controller that preserves object stability. The latter adjusts gripping force in response to detected slippage on site that may be caused by factors such as inadequate initial grip force or external disturbances. Kyberd and Chappell (1994) pioneer developments in this field, proposing a hierarchical control system, with force feedback provided by an electro-optical system. Testing conducted with an amputee demonstrated performance levels comparable to those achieved with the user’s regular prosthesis (Kyberd et al., 1993). Other examples employed inertial measurement units (Arapi et al., 2020) or optical sensors (James et al., 2018) for slip detection. Among popular slip detection techniques, the friction cone method relies on measured normal and shear forces, while vibration detection involves bandpass filtering of the raw pressure signal. In a comparison study by Reinecke et al. (2014)), the friction cone method demonstrated accurate slip detection robust to both impact and motion. However, its implementation complexity required prior grasp knowledge to develop an accurate model of the object or grasp. In contrast, bandpass filters offer a faster reaction time but are prone to disturbances.

Furthermore, there is a growing interest in using machine learning methods to overcome the simplifications required by conventional analytical approaches. In Kwiatkowski et al. (2017)), a grid of pressure sensors together with proprioceptive information of a gripper was employed to train and evaluate multiple convolutional neural networks. Similarly, Funabashi et al. (2021) detected slip and deformation using multilayer perceptions with optical sensors capable of measuring both normal and shear forces. Nonetheless, when compared with simpler approaches, results from Reinecke et al. (2014) suggested longer reaction times and higher computational costs for machine-learning-based methods.

This work introduces biomimetic low-level controllers aimed at mimicking human reflexes to enhance the intuitiveness of control strategies. Healthy individuals effortlessly grasp objects, with the high-level goal of grasping being decided by the subject, while the grasp geometry selection and pre-shaping of the hand occur with minimal conscious effort. Accordingly, the proposed solution integrates tactile information, gathering both normal and shear forces, to autonomously guarantee grasp stability, while ensuring user authority. Based on prior findings, this work proposes a hybrid slip detection method combining the robustness of the friction cone model with vibration detection for initial model creation. Experimental validation was conducted and the results were compared with existing methods. The first experiment involved grasping five objects with a sensorized robotic hand and applying impulsive forces to induce slip. Moreover, a qualitative validation demonstrated the similarities with human behavior while lifting an object and reacting to unexpected weight changes.

## 2. Materials and methods

To address the limitations of existing slip control methods, this work proposes a combined approach for shared autonomy inspired by human physiology. Then, we evaluate the effectiveness of the proposed approach in a robotic hand with tactile sensors and compare the performance with existing methods. Four different control conditions were implemented and tested: no controller, bandpass filtering, friction cone, and the proposed combined controller. During the experimental validation, we consistently induced three slip events in multiple objects grasped by the robotic hand. Moreover, a qualitative test showed similarities with human behavior.

### 2.1. Combined slip control for shared autonomy

**Figure 1.**
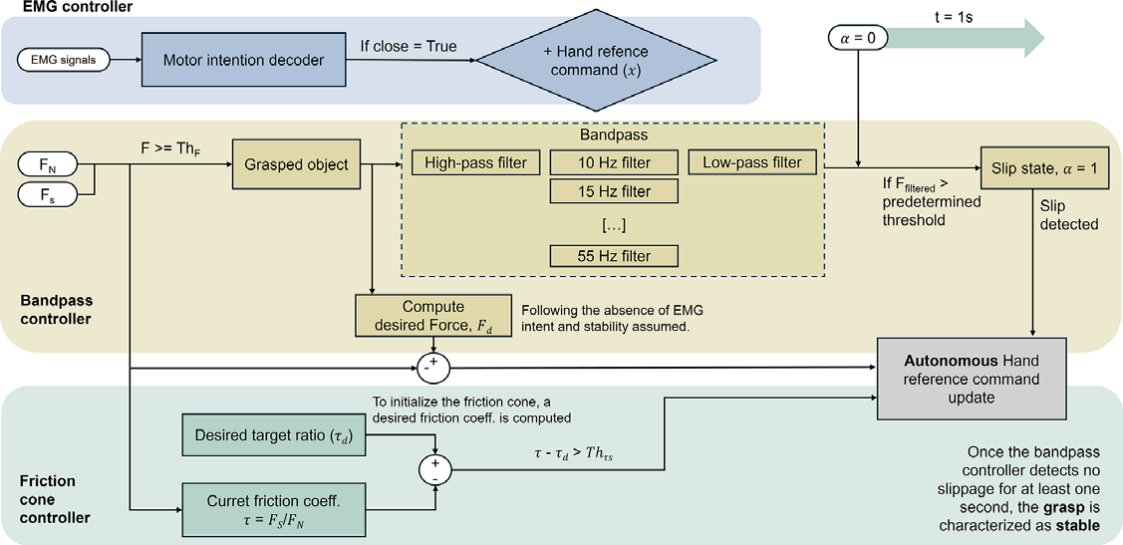
Control workflow of the proposed strategy. The three integrated controllers are distinguished by different background colors, with measured EMG signals, normal forces ( 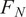 ), shear forces ( 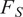 ), and the initialized 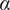 state shown in white. The EMG controller represents the high-level command used to set the hand reference value, while the bandpass and friction cone controllers operate within the low-level decision-making process to enable autonomous hand behavior.

The proposed shared-autonomy control approach entails three control states: EMG-based control, bandpass control, and friction cone control (Figure 1). The friction cone method is noted for its rapid response time and resilience to external disturbances, making it suitable for real-life scenarios faced by prosthetic users. However, its model relies on parameters such as surface texture and object characteristics (Reinecke et al., 2014), limiting its practicality for everyday use, especially in unknown conditions. In contrast, the bandpass method is simpler to implement but more sensitive to external disturbances. Accordingly, we propose a slip control method that involves detecting initial slip based on high-frequency vibrations from grasping forces and maintaining grasp stability through an appropriate shear and normal forces ratio.

The main state from the proposed shared control involves processing EMG signals to command hand opening and closing, prioritizing user voluntary control. Here, we propose a traditional on/off direct control scheme. If the EMG signals from the agonist muscle group surpasses a predefined threshold without simultaneous activation of the antagonist muscle group, the robotic hand will either close or open at a constant speed. In the absence of EMG input, while contact is confirmed, the controller switches to a low-level slip control, initially employing bandpass filtering for stability. Initially, the offset of the sensors is removed by referencing the first five samples of each trial with no movement artifact. Subsequently, the corrected shear force data are fed into the bandpass slip detection module, resulting in an input to each filter in parallel. When a stable grasp is confirmed, the algorithm transitions to the friction cone control module, leveraging its robustness without requiring a preset model. The latter is achieved by defining the desired ratio between the shear and normal forces immediately after an initial slip event. When this event has been corrected through the bandpass controller, and assuming a stable grasp afterward, the ratio is computed.

#### 2.1.1. Friction cone controller

The friction cone controller represents a slip detection method that focuses on the continuous concept of the friction cone. Friction is the force resisting relative motion between at least two solids. While a complex phenomenon influenced by factors such as surface deformation, friction can be simplified using Coulomb’s Law: 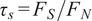 , where 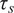 represents the static friction coefficient, defined as the maximum ratio of shear force ( 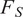 ) to normal force ( 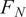 ) before movement initiates. The friction coefficient is frequently employed in robotics to assess grasping safety. In particular, the friction cone controller is proposed in Reinecke et al. (2014)) as a virtual cone formed by the vertex of a contact point between the end-effector and the object surface, along with the friction angle that prevents movement ( 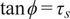 ). Stability in grasping is determined by whether contact forces remain within the friction cone boundaries, indicating a stable grasp, or surpassing them, suggesting slippage.

A biomimetic sensor, proposed in Wettels et al. (2008), operates as a variable impedance sensor that uses the displacement of a fluid between an elastomeric skin and electrodes to measure both normal and shear force. In Wettels et al. (2009), this sensor was placed on the thumb of a commercial 2-DoAs hand to continuously compute the ratio of shear to normal forces and compare them against a predetermined optimal estimate, named 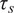 . When this ratio exceeded a set threshold ( 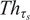 ), the hand adjusted its reference command by a predefined amount. Experimental outcomes demonstrated successful prevention of object dropping, with failure only occurring with the fastest force perturbations. In Song et al. (2013), a more sophisticated LuGre model was used to compute a real-time critical ratio 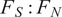 , employing force/torque sensors analyzing both object acceleration and forces. However, this latter approach was limited to specific surface types and required experimental determination of friction parameters for the model.

Within the proposed methods, once the bandpass controller detects no slippage for at least 1 s, the grasp is characterized as stable, prompting a transition to the friction cone control state. To initialize the friction cone, the desired friction coefficient, 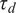 , is computed, using five force samples window to mitigate the influence of sensor noise. Then, the friction cone technique is guaranteed through the implementation of a PI controller, defined by:
(1)

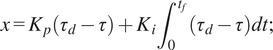

with 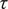 being computed in a five-sample window and using the average normal and shear force considering all tactile sensors. This controller aims to maintain the ratio between average shear and normal force near the target ratio ( 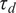 ), preventing excessive squeezing when the ratio decreases and slip occurrence when the ratio increases.

#### 2.1.2. Bandpass controller

Robotics has adopted anti-slip strategies that detect slip by sensing high-frequency vibrations produced by friction, similar to human reflexes mechanisms. Typically, force-sensitive resistors have been placed at the fingertips of artificial hands to identify slippage by analyzing fluctuations in the exerted normal forces (e.g., Pasluosta et al., 2009; Pasluosta and Chiu, 2012). The control algorithm traditionally comprises various stages, including force control and slip detection. Upon detecting a slip, the force reference is increased. Although neural network algorithms have proven effective for slip detection and mitigating limitations associated with inexpensive sensors, their high computational costs restrict their widespread application in real-time controllers. In a more simplistic approach, the derivative of normal forces has been used to create a binary slip signal by thresholding (Gentile et al., 2020). This slip signal, integrated over time, was included in the error functions for finger positions, subsequently driven to zero by a controller that predetermines the desired force. Moreover, the analysis revealed that high-frequency components in the derivative of shear force detected through strain gauges are indicative of slip occurrences, with different slip events displaying distinct frequency components (Engeberg and Meek, 2011). Experimental results demonstrated that the proposed integral sliding mode controller yielded minimal object deformation while ensuring object retention, thereby enhancing slip detection efficiency.

The slip detection algorithm implemented in this work relies on bandpass filtering of the input signals introduced in Engeberg and Meek (2011). Each shear force signal undergoes individual filtering using a fifth-order digital filter, consisting of three main stages. A first high-pass filter is applied to remove low-frequency components. Then, the signal undergoes two rounds of filtering by a second-order bandpass filter, targeting resonance near 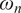 . Implementing multiple bandpass filters at distinct frequencies enables the detection of slippage using a simple threshold approach. The bandpass filter was set up to operate in parallel at 10 distinct frequencies ranging from 10 to 55 Hz, with increments of 5 Hz. If any of the filtered signals exceed the predetermined threshold, the variable slip state ( 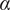 ) is equal to 1; otherwise, 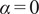 . Different thresholds were applied for each resonance frequency. Finally, a low-pass filter is employed to reduce high-frequency noise.

**Figure 2.**
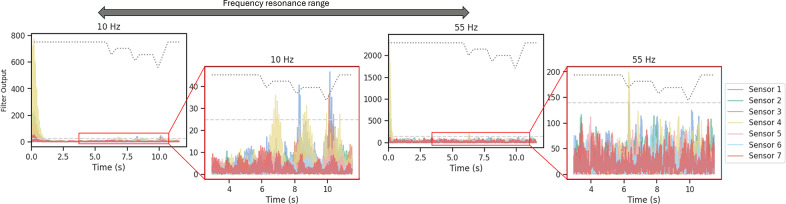
Bandpass filtering output for the sensor in the index finger. This data corresponds to a trial involving a small cylinder with an initial closure of 30%. Each resonance frequency is plotted along with its corresponding threshold (dashed line). Panel (a) shows the signal for the entirety of the trial. Panel (b) is zoomed in to the time interval where slip is induced.

Figure 2 illustrates the output of the filtering method employed for slip detection during a trial. The plot displays the filtered signal for the y-axis of force at the lowest and highest bandpass resonance frequencies, along with their respective thresholds. Notably, slip events can exhibit distinct frequency components. While the 10 Hz filter successfully identifies all three slip events, the 55 Hz filter can only detect the first one. However, it detects the start of the slip event 0.458 s earlier than for the 10 Hz filter. Despite effective identification of all three slip events through thresholding, the initial signal instances are also characterized by high amplitudes, potentially leading to misclassification as a slip event.

Regarding the force control stage, we compute the hand reference command ( 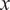 ) employing a PI controller, using overall force measurements and slip state binary signal ( 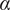 ). This controller is expressed as:
(2)

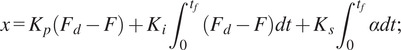

where 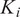 , 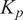 , and 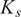 denote the integral, proportional, and slip gains, respectively. 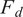 represents the desired force and is defined upon initiation of the shared controller, following the absence of EMG intent and stability assumed. 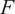 is the measured force, computed as the vector norm of the total gripping force from all tactile sensors. The last component of the equation is the slip error, which quantifies the occurrence of slip. By integrating the slip state ( 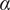 ), a steady increase in the hand reference command is ensured within slip events. Note that the desired force 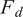 remains unchanged. Therefore, once external disturbances stop and 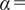 0, the hand goes to the original geometry.

### 2.2. Experimental setup

**Figure 3.**
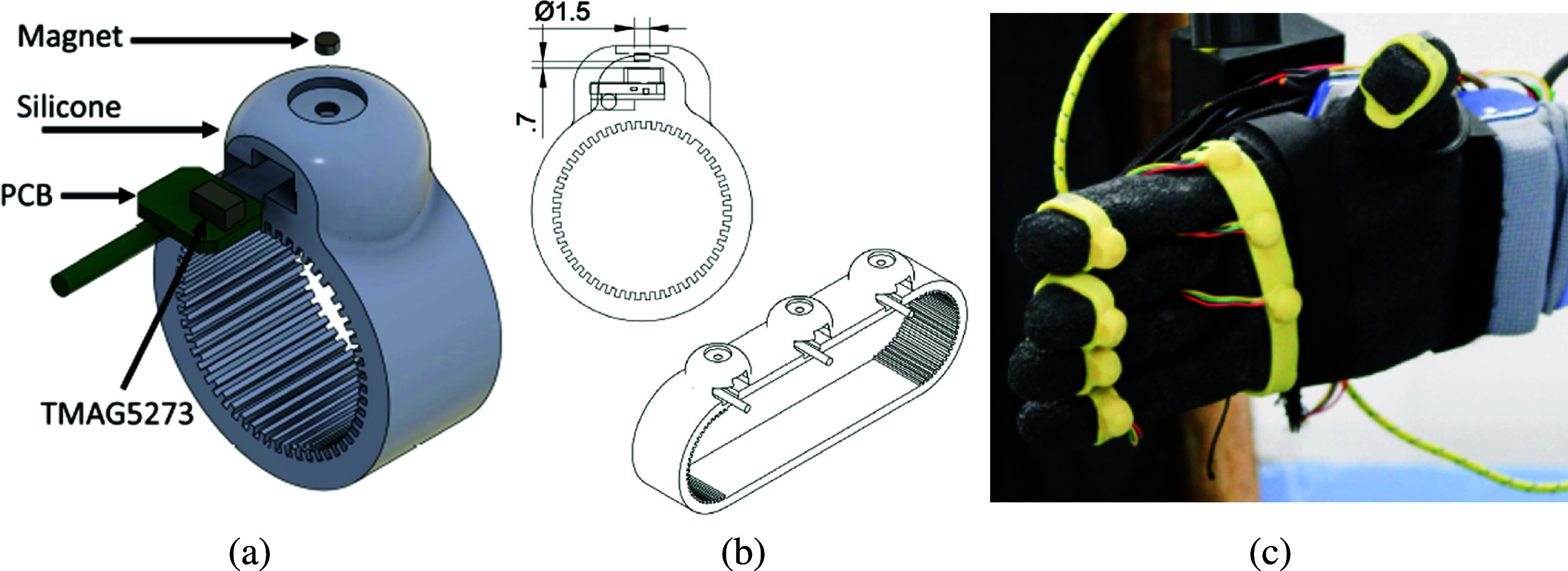
Designed Hall effect force sensors. Panel (a) shows the exploded view of the ring sensor. This includes a magnet, silicone cover, PCB, and IC. Panel (b) reports the placement of the magnet located 0.7 mm above the IC and the band design used for the upper palm area. Panel (c) depicts the final sensor placement within the robotic hand.

**Figure 4.**
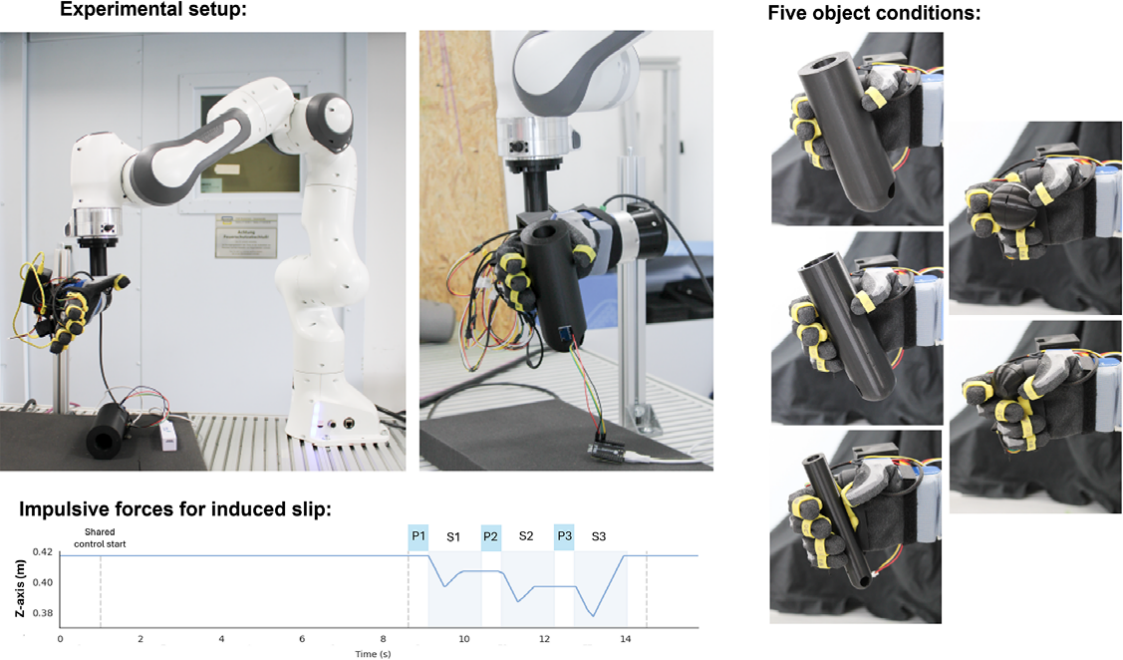
Experimental protocol. The top panel shows various components of the setup and the relative location of the Panda robotic arm with respect to the hand and grasped object. The central panel visualizes the objects used in the experiment, corresponding to the tripod, pinch, and cylindrical grasping actions. The bottom panel reports the trajectory of the end-effector for impulsive forces. The pre/post-impact intervals are indicated with the letter P, while the intervals where the impact happens are marked with the letter S.

In this work, we implemented soft-embedded Hall effect sensors to retrieve force measurements. Each designed force sensor integrates a TMAG5273 magnetic sensor (Texas Instruments, USA) comprising three Hall effect sensing elements (Ramsden, 2011), enabling measurement of magnetic flux along three axes, observing both normal and shear contact forces. The sensor features an integrated analog-to-digital converter with 12 bits of resolution (12-bit ADC), offering various configurations including two magnetic ranges. For this setup, ranges of 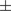 40 mT for the *x* and *y* axes and 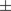 80 mT for the z-axis were chosen, with temperature compensation set at 0.12%/°C. The chosen update rate corresponds to averaging every 32 samples, ensuring an optimal signal-to-noise ratio. An N45 disc magnet with axial magnetization (diameter = 1.5 mm, height = 0.5 mm) is positioned 0.7 mm above the sensing elements in the integrated circuit (IC), serving as the force-transducing component. The magnet and IC are encased within a silicone shell (Dwivedi et al., 2018), with a shore hardness of 35A, chosen based on prior experiments and its resemblance to typical silicone prosthetic skins (see Figure 3). Ring-shaped design for insertion into the fingers and band-shaped for placement in the palm were developed, as introduced in Castañeda et al. (2023). To enhance friction between the silicone and the hand surfaces, thereby mitigating sensor rotation, the inner surface of the silicone was textured with ridges. As force is applied to the silicone dome, the relative position between the magnet and the IC changes, leading to variations in the magnetic field, which the IC outputs as a digital signal. Since each sensor was handcrafted, it exhibited slight variations in its physical characteristics, leading to unique relationships between magnet displacement and applied force. Consequently, individual sensor calibration was necessary to convert the magnetic flux density into force measurements. Seven sensors are used for this study, five in the form of rings and a band embeeding two of them centered in the palm. The sensors are commanded with a microcontroller and powered by a battery, housed in a 3D-printed box (see Figure 4).

The sensors are integrated into a robotic hand, the qb SoftHand Research 2 (qbrobotics, Italy). This is an anthropomorphic hand with 19 degrees of freedom (DoFs) and equipped with two actuators and a single tendon. Its design adheres to the principles of soft robotics, employing compliant rolling-contact joints interconnected using elastic ligaments (Della Santina et al., 2018). The placement of the sensors within the robotic hand was determined by our previous investigation (Castañeda et al., 2023) on the spatial and temporal distribution of forces during daily activities in humans. This research highlighted the significant contributions of the fingertips and thenar eminence to grasping forces in both shear and normal directions. However, the selected robotic hand employs a rigid palm, without a dedicated joint to mimic the human thenar eminence and does not conform to the grasped object. Consequently, five sensors were allocated to the fingertips of the robotic hand, with additional two placed in the upper palm area (Figure 3[c]). With the current setup and configuration, a sampling frequency of approximately 200 Hz was achieved. The sensorized robotic hand was mounted onto an adapter connected to an aluminum profile, securely fixated at the wrist and suspended horizontally above the table.

To induce slip events reliably across trials and control schemes, we employed a 7-DoFs robotic arm (Franka Emika, Germany) to press abruptly the top surface of the grasped objects. This robotic arm boasts velocities of up to 2 m/s at the end effector and offers a precision of 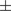 0.1 mm. A cylindrical end-effector was 3D printed and attached to the robotic arm (see Figure 4). Additionally, we integrated an IMU (MPU-6050 - TDK InvenSense, USA) onto the grasped object to estimate its displacement via acceleration. The IMU data were sampled at approximately 330 Hz.

### 2.3. Induced slip experiment

For this experiment, an object was manually placed within the robotic hand before initiating hand closure. When the object is gripped by the robotic hand, the robotic arm descends by 2 cm from the initial position (see Figure 4) to impact the object with its cylindrical end-effector. It then retracted by 1 cm before pausing for 1 s. Note that the initial position of the robotic arm was above the grasped objects to minimize vibrations caused by motion artifacts. This sequence was repeated for each object, ensuring impulsive forces were applied to the object three times per trial, as depicted in Figure 4. Each slip event was divided into three distinct time intervals: pre-impact (P phase), during impact (S phase), and post-impact (P phase), with the post-impact interval aligned with the pre-impact phase of the subsequent event. Four controllers were tested for each experimental condition. For the *no controller* case, the hand closure level remained constant throughout the trial, even though slippage occurred. For the *bandpass controller*, only the controller detailed in Section 2.1.2 was activated. The *friction cone controller* adopts the approach described in Section 2.1.1, obtaining the friction model immediately after the hand reaches its targeted reference command. The *combined controller* implements the proposed architecture described in Section 2.1, which integrates the bandpass and the friction cone methods. Five objects were designed and 3D printed to evaluate distinct grasping conditions, comprising three cylinders (diameter = 60, 50, 25 mm), more suitable for power grasps, and two balls (diameter = 55, 40 mm), representative of a tripod grasp and a pinch (see Figure 4). To ensure consistency across trials, the EMG module was replaced with direct commands for targeted hand reference command (also named initial closure level), which was treated as a factor in the statistical analysis. Three different initial closure levels were introduced as experimental conditions: 20, 25, and 30%. The 20% closure was excluded for both small objects due to insufficient contact. Each experimental condition underwent five repetitions, resulting in a total of 260 trials and 780 slip events analyzed.

### 2.4. Qualitative validation for human similarities

**Figure 5.**
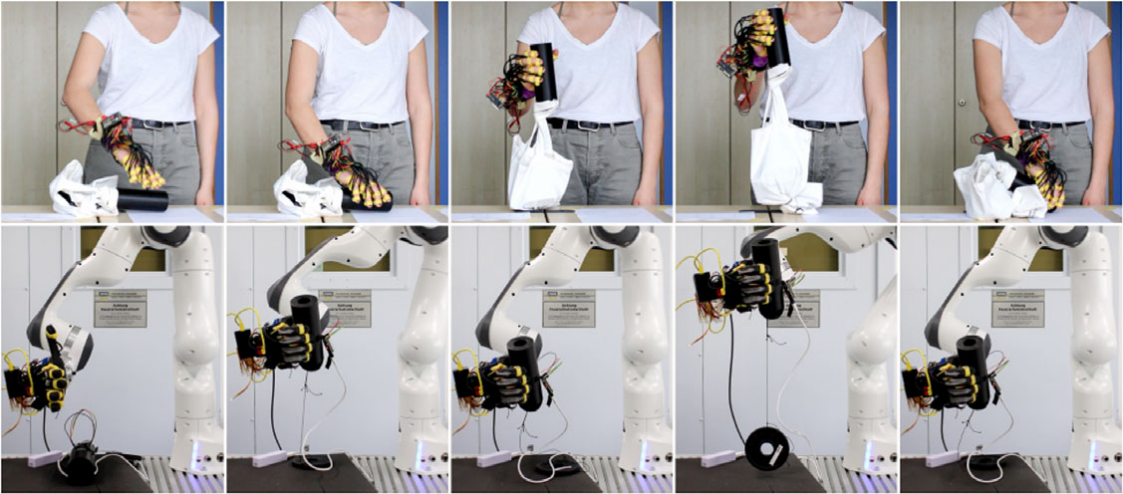
Human behavior comparison. Photo sequences exemplify the protocol for the human study (top row) and for its comparison with the shared-autonomy control (bottom row).

Given the inspiration drawn from human physiology to design the proposed controller, we conducted experiments in Castañeda et al. (2023) using the same sensors placed in human hands to collect tactile data. Force measurements were gathered from six human participants (age 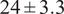 years, all male) grasping a large cylinder using their dominant hand. The cylinder was connected to a bag with an unknown weight inside. Upon achieving a stable grasp, participants were instructed to lift the cylinder until the weighted bag attached to its base was completely raised from the table (see the top row in Figure 5). Unaware of the weight inside the bag, participants adjusted their applied force based on sensory feedback and weight estimation to prevent object loss. For this purpose, their hands were equipped with 20 force sensors, with one sensor placed in each phalanx and six in the palm region. This experimental procedure was repeated with three weights (0, 1, and 3 kg).

To replicate this behavior with the robotic hand and evaluate various slip controllers, we adapted the previous experimental protocol. To recreate a similar scenario, a 0.5-kg weight was fixed to the large cylinder using a nonelastic string. The robotic hand was directly attached to the robotic arm as the end-effector, with the thumb pointing upward (see bottom row in Figure 5). The EMG module of the shared control was replaced by a targeted hard reference command. The cylinder was placed within the hand, and an initial closure level of 20% was commanded. The robotic hand was then raised by 20 cm to ensure full suspension of the weight, expecting the initialization of the autonomous slip control. After a 2-s hold, it returned to its original position. This protocol was conducted once for each of the four controllers as a qualitative validation. Following the methodology employed in Castañeda et al. (2023) for human recordings, time is normalized between 0 and 1, and sample synchronization is conducted based on the trajectory of the robotic arm. A qualitative assessment of the temporal evolution of grasping shear forces allowed a preliminary validation of similarities between both experiments and controller behaviors.

### 2.5. Data analysis

To evaluate the performance of the slip detection algorithm, the sensor data were segmented into the S and P intervals, as presented in Figure 4. To assess the effectiveness of the various controllers, three primary metrics were chosen: average and maximum force, and maximum acceleration, computed for each of the pre-impact (P) and slip (S) intervals. We expect detection of slip during S intervals, whereas P intervals should be characterized by no slip. Data distributions from these metrics underwent a normality check though a Shapiro–Wilk test. As data follow nonparametric distributions, the Friedman test was employed with statistical significance determined based on right-tailed chi-square critical values. When a factor is significant, a post-hoc pairwise analysis is conducted using the Wilcoxon test with Bonferroni correction.

## 3. Results

**Figure 6.**
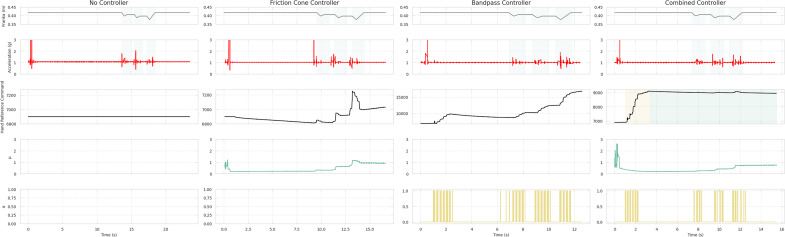
System response and slip detection. From left to right column, (a) panels report the controller outputs for the no-controller case. Although there is significant acceleration, the hand aperture remains constant. Panel (b) shows the system response with the friction cone controller. Panel (c) presents data for the case with the bandpass controller. The disturbances are detected by the frequencies algorithm, resulting in an increase in hand reference command while slip occurs. Panel (c) presents data for the case of the proposed controller, combining both methods. Slip is initially detected using bandpass filtering. The controller then switches to the friction cone method, where increases in the friction coefficient ( 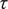 ) induce an increase in the hand reference command.

Figure 6 illustrates the system’s behavior with all tested low-level controllers, depicting a trial involving the small cylinder with an initial closure of 30%. The initial acceleration peak observed is attributed to the hand initial closing or other movement artifacts. The first column shows data for the *no controller* case. Disturbances caused by the robotic arm (impulsive forces) were uncorrected due to the absence of slip control, leading to a consistent hand closure reference command throughout the trial. The second column shows the response to the *friction cone controller.* As for the other cases, the initial hand closure prompts a peak in acceleration and a high friction coefficient. To counteract this 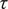 , there is a gradual reduction in the hand reference to preserve 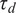 . This behavior lasts until the first slip event is detected. Disturbances generated by the robotic arm result in the rise of the friction coefficient and a corresponding proportional increase of the hand reference command. However, as the friction coefficient value remains higher than the set value after the slip events, the hand closure continues to increase by the end of the trial. The response of the system testing the *bandpass controller* is depicted in the third column. The initial acceleration peak leads to perceived instability and a slip signal 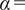 1, prompting an increase in the closure reference command until a stable grasp, defined as the instant without detected slippage. Subsequently, the hand reference command gradually decreases as the controller endeavors to reach the target force initially set upon activation of the bandpass controller. When the robotic arm interacts with the object, generating disturbances, slip is successfully detected in three main intervals. Consequently, the hand reference command increases according to a preestablished gain. Note that the third slip event corresponds to a higher acceleration, prompting a larger and more pronounced increase in grasping forces. Finally, an exemplified response of the *combined controller* is reported in the fourth column. Initially, there are peaks in acceleration and friction coefficient due to movement artifacts. Upon start of the slip control method, within the bandpass controller phase, slip is promptly detected, leading to a rapid increase in closure level. Although the friction cone controller is inactive at this stage, resulting in no dependence on this parameter, a decrease in the friction coefficient can be also observed. After these initial increases and a lapse of 1 s without slip detected, the bandpass controller is deactivated, and the friction cone controller is engaged in a second phase. As the robotic arm induces impulsive forces on the object, the friction coefficient rises, prompting an increase in hand reference command sufficient for slip avoidance.

**Figure 7.**
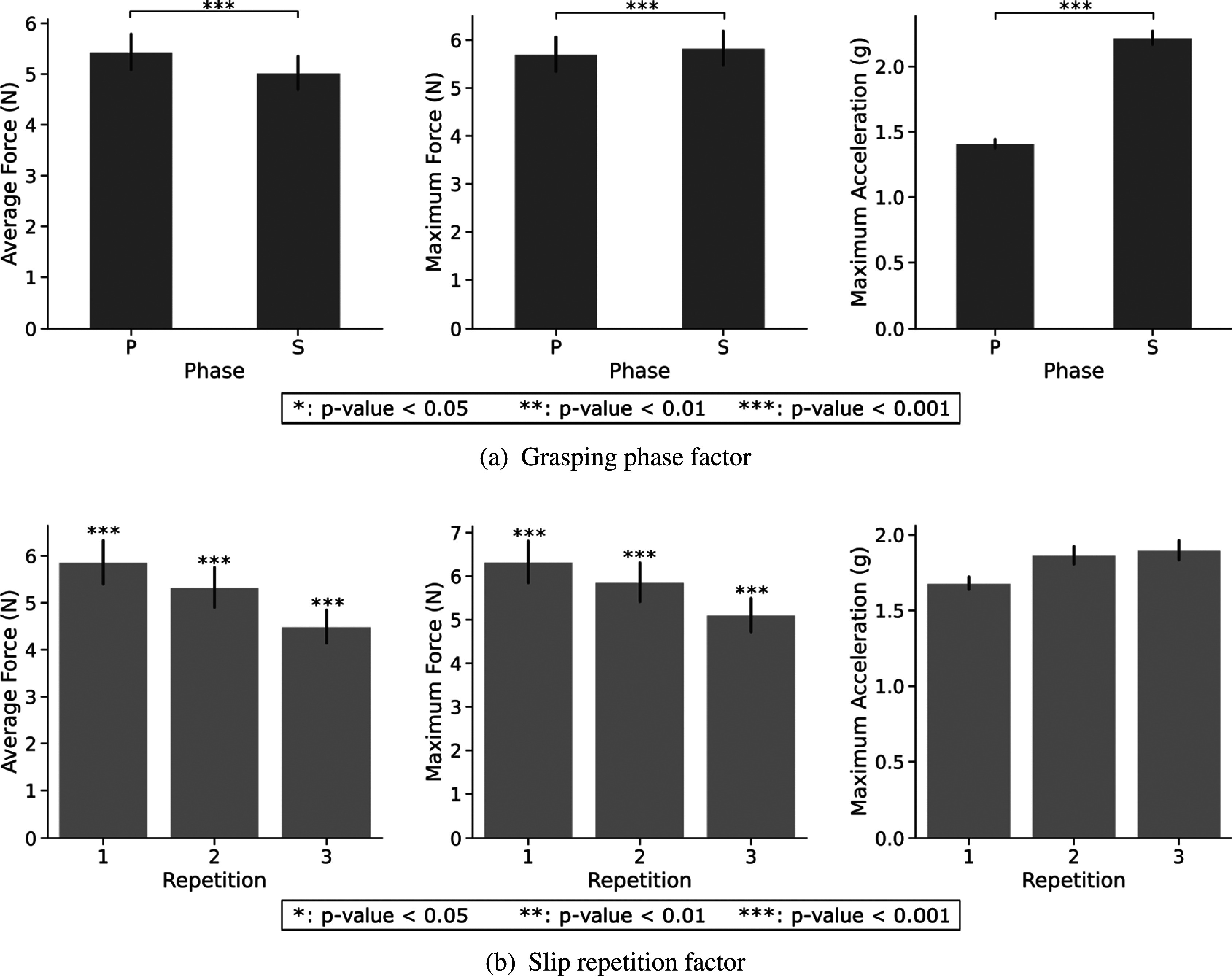
Performance metrics for grasping phase and slip repetition factors. Data displays all controllers, objects, and initial closure levels.

To evaluate the effectiveness of the various controllers, three performance metrics were assessed: average and maximum force, and maximum acceleration. Results from the Friedman test considering five factors are reported in Table 1. A Wilcoxon test with Bonferroni correction was applied to observe differences within conditions of a significant factor. Post-hoc significance is reported exclusively in the format of asterisks within the figures. Note that both *Phase* and *Repetition* present significant differences among conditions for the three performance metrics, except for the acceleration peak in the slip repetition factor. Results from these two factors are depicted in Figure 7. Phase results demonstrate larger maximum forces and accelerations during the S phase, while slightly larger average forces are applied during the P phase. On the other hand, repetitions achieved lower applied forces with more slip repetitions, while maximum acceleration is consistent within the slip event interval.

**Table 1.** Results of the Friedman test using controllers, objects, initial closure level, phase, and repetition as within-subject factors

	Average force		Maximum force		Maximum acceleration	
	Q	p-value	Q	p-value	Q	p-value
Controllers	123.35	<.001	101.49	<.001	16.54	<.001
Objects	132.85	<.001	138.42	<.001	10.78	.029
Closure	80.11	<.001	77.19	<.001	2.25	.325
Phase	23.08	<.001	17.33	<.001	74.77	<.001
Repetition	67.46	<.001	41.21	<.001	3.00	.223

*Note*: Q is the Friedman chi-square statistic.

**Figure 8.**
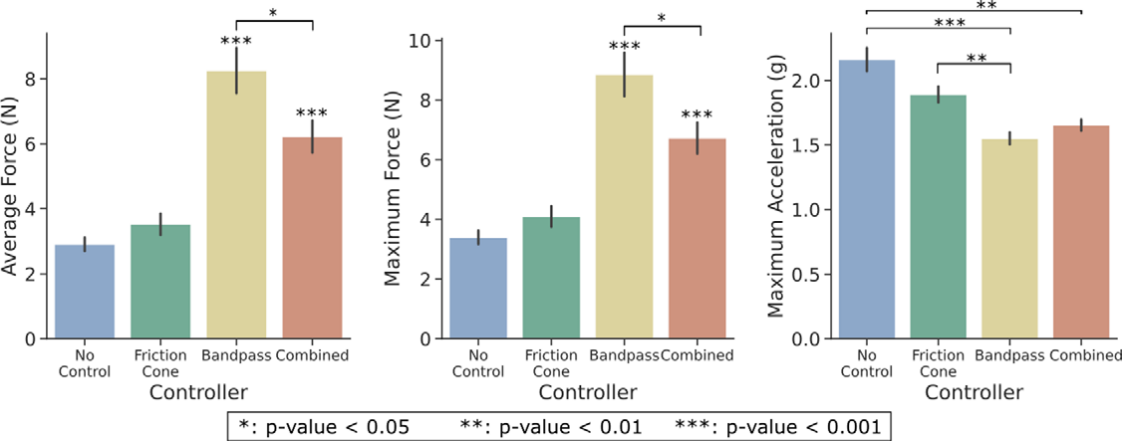
Performance metrics for each controller. These data consider all objects and initial closure levels.

Significant differences are also observed for the different *Controllers* for all three metrics, assumed based on the right-tail chi-square critical values. The controller outcomes are depicted in Figure 8. The pairwise examination reveals significant differences among all controllers for both force metrics, except for the no-control friction cone pair, with lower applied forces to the object. However, this pair reported larger maximum acceleration values with respect to bandpass and combined controllers. Between the combined and bandpass controller pair, the latter achieves a significantly higher grip force. Note that the maximum force metric obtained very similar results to the average force, with approximately 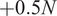 . Regarding the maximum acceleration, no significant difference was observed for bandpass-combined pair, even though no control only showed a significant difference in acceleration values with respect to the combined controller.

**Figure 9.**
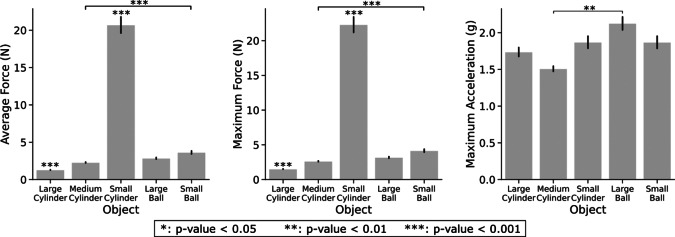
Performance metrics for each object. The data displayed considers all initial closure levels and controllers.

**Figure 10.**
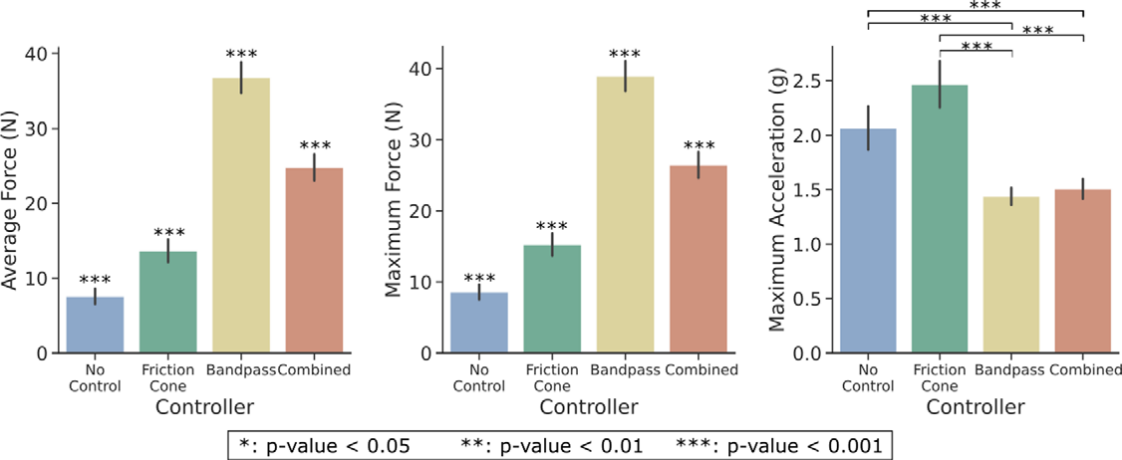
Performance metrics for each controller in small cylinder trials. These data account for all initial closure levels, but only for the small cylinder trials.

Figure 9 reports controller results categorized by the properties of the *Object* grasped. The Friedman test indicates object dependencies for all three performance metrics. Post-hoc analysis reveals significant differences among objects consistent for both force metrics, with the large and small cylinders being significantly different from the other objects, and with the lowest and largest forces applied. Moreover, the medium cylinder showed significance with respect to the small ball. Regarding maximum acceleration, the corrected Wilcoxon test indicates significant differences only between the medium cylinder and the large ball. Similar trends to Figure 8 with higher significance between pairs are observed in controller outcomes for the small cylinder trials, reported in Figure 10. The average and maximum force exhibit a progressive increase in the following order: no controller, friction cone, combined, and bandpass controller. Furthermore, the bandpass and combined controllers achieved the lowest value for the maximum acceleration (approximately 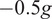 with respect to no-control friction cone pair), as seen in Figure 10.

**Figure 11.**
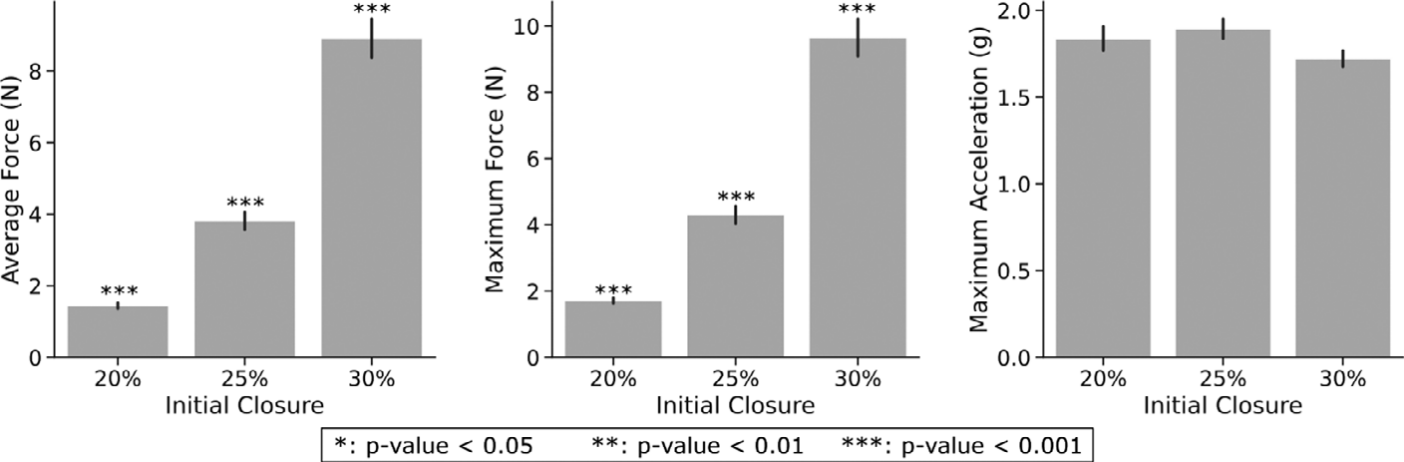
Performance metrics for each initial closure. The data displayed consider all objects and controllers.

Another independent variable impacting performance is the initial closing level (*Closure*) of the hand, shown in Figure 11. However, according to the Friedman test (Table 1), this factor does not yield significant differences within conditions for the maximum acceleration metric. The pairwise comparison indicates significant differences among all pairs for both force metrics with higher forces consistently associated with larger targeted hand reference commands and a more closed hand.

**Figure 12.**
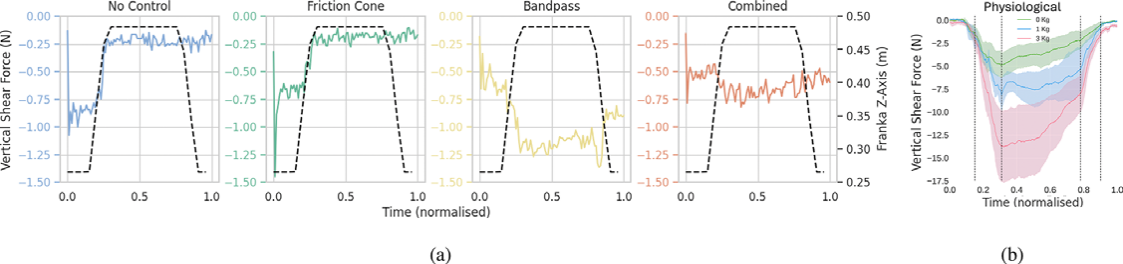
Shear force of a lifting trial for each of the controllers (a) and the human study (b). The dashed lines in (a) describes the robot trajectory, while in (b) highlight grasping phases instants where changes in shear forces are observed.

Finally, a qualitative human study was conducted to compare reaction forces with the controller behaviors. The shear force data from the human comparison trials is reported in Figure 12, including the physiological controller. Both the no control and the friction cone controller failed to complete the task, leading to the object being dropped when the tendon attached to the weight was fully extended. This is evident from the sudden drop in shear force observed in both plots as the hand was lifted. Conversely, both the bandpass and combined controllers successfully lifted the object by adjusting the grip force, as indicated by the prompt increase in shear force as the weight of the object is perceived.

## 4. Discussion

Our results showed that the initial slip detection strategy effectively monitored filtered shear sensor output for each resonance frequency in parallel through a simple thresholding. Nonetheless, during the initial grasp stages of the trials, signal amplitudes were higher than those during induced slip events, possibly influenced by residual setup movement or vibrations from the robotic arm. For instance, slip ( 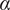 ) is detected (from *t* = 1–3 s) as the robotic arm moves to its initial position, as shown in Figure 6. To mitigate this effect, the use of upper thresholds was suggested in Teshigawara et al. (2011). However, this could compromise the algorithm awareness of the actual slip event duration. The sensitivity of this method to external disturbances, also noted in Reinecke et al. (2014), emphasizes the importance of careful frequency selection and threshold adjustment, especially considering variations in object shape and size.

Regarding the controller performance comparison, this sensitivity also affected the response of the bandpass and combined controllers, misinterpreting movement artifacts as slip events. Consequently, these controllers applied larger initial forces compared to the friction cone controller (Figure 6). In lab settings, the slip should be measured based on displacement variations. However, due to challenges in directly measuring object position, the acceleration from an IMU was employed instead. While theoretically, acceleration could be used to compute displacement, the presence of noise in IMU measurements rendered this approach impractical. According to the selected performance metrics, the combined controller successfully adapted the grasping force (or hand reference command) as slip is detected, resulting in lower acceleration peaks compared to no control and friction cone controller, and in a significant reduction in forces with respect to the bandpass controller (Figure 8).

**Figure 13.**
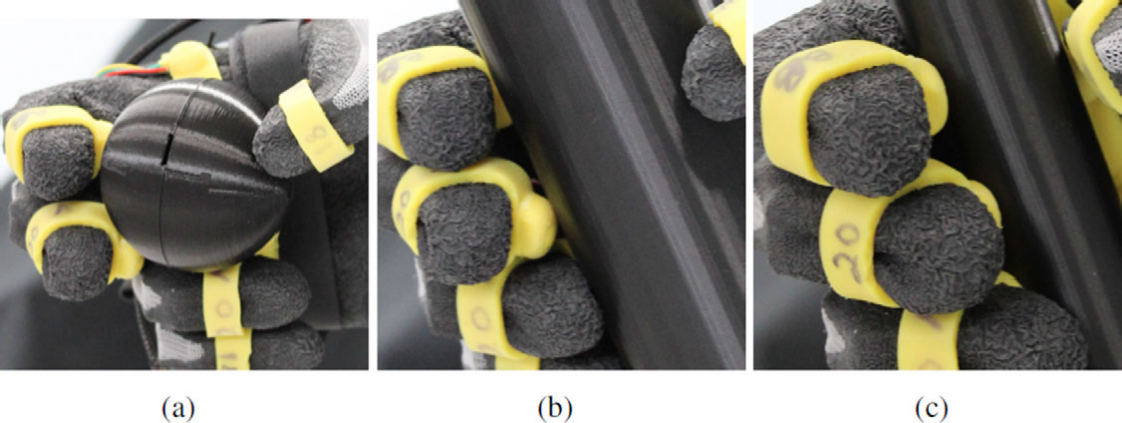
Examples of contact between the object and sensors. Panel (a) shows insufficient three-finger contact with the large ball, and panel (b) for five-finger contact with the medium cylinder. Panel (c) shows better contact achieved with the small cylinder.

Furthermore, diminished performances in slip detection during induced slip events can be attributed to the somewhat bulky design and protrusion of the developed tactile sensors, particularly noticeable under certain experimental conditions. The sensors are designed to yield optimal force signals when making contact with the object directly above the center of the embedded magnet, perpendicular to the sensor’s flat surface. Nonetheless, achieving precise control over hand final geometry poses challenges due to its soft properties and the tendency of anthropomorphic grasping to make contact from varying angles (Wu and Santello, 2023), leading to suboptimal sensor contact. Figure 13 showcases this issue, with sensors in the thumb and middle finger failing to establish perfectly perpendicular contact with the large ball or medium cylinder surface. Similarly, Figure 9 demonstrates that while the median cylinder and large ball exhibit equivalent forces, they present significantly different slip scenarios, indicating potential variations in grasp geometry or failure to achieve optimal sensor contact. Likewise, the large and small cylinders exhibit the most disparity in applied force among objects, with the small cylinder registering the highest. However, both objects report very similar acceleration peaks, associated to grasp stability. Therefore, we hypothesize that larger measured forces between objects solely indicate higher contact quality. The initial closure level could also influence the algorithm performance, with both insufficient and excessive hand closure adversely affecting slip detection quality. For instance, findings from Figure 6 illustrate that an increase in the number of grasp repetitions correlates with lower measured force values. Nevertheless, the reference command tends to be higher with repetitions due to slip corrections, suggesting potential issues with sensor contact and the accuracy of measured forces in representing the actual grasp force.

The superior performance of the combined controller was especially visible under the small cylinder trials, partially attributed to better sensor contact and the effectiveness of slip detection for this object. Therefore, the importance of establishing optimal contact between sensors and objects extends also to the distinct control states and their effectiveness. Accurate force measurements are essential for both the force and friction controllers implemented within the proposed method. Accordingly, the results obtained with the small cylinder provided a clearer illustration of the underlying principle behind the combined controller, due to a higher contact quality, with respect to the state of the art. For this object, both the average and maximum force exerted by the combined controller were considerably lower than those generated by the bandpass controller. As expected, the friction cone and no control resulted in even lower forces. However, these two controllers yielded much higher object accelerations than the combined and bandpass controllers, indicating a greater amount of slip induced during the trials. Considering both force and acceleration results, the proposed combined controller represents a good trade-off between appropriate grasping force and object slip avoidance. Nonetheless, optimizing sensor contact and grasp force values is imperative for developing a real-time controller that ensures grasp stability.

The proposed approach was preliminarily validated in a human study comparison, where both the combined and bandpass controllers successfully adapted the grasp force, and the object was lifted. In contrast, the no controller and the friction cone controller failed to maintain stability, leading to the object being dropped when its weight increased. This failure highlights the limitations of assuming stability immediately after the hand closes, suggesting an inadequate friction model from the user point of view. Furthermore, while the exact response time from disturbance creation to correction initiation was not determined, the bandpass slip detection took roughly 16 ms. An intentional delay was introduced and associated with a five samples windows for friction coefficient calculation, resulting in an approximately 25 ms delay. Thus, the combined controller meets the requirement of system operation at a maximum latency of 70 ms (Zangrandi et al., 2021) for biomimetic robotic tactile control systems. Another criterion for this is to minimize automatic force gain slightly above the level necessary to prevent slip. Although the combined controller succeeded in reducing grasping force compared to the bandpass controller, preferably for the manipulation of common objects and artificial hand usage, further adjustments to slip thresholds and force gains could enhance the system performance.

## 5. Conclusions

This work introduces a novel hierarchical controller for myoelectric hand prostheses inspired by human hand physiology and sensory reflexes in grasping. This approach integrates a traditional EMG high-level controller with the combination of two low-level solutions for slip control. In particular, it bridges the robustness of the friction cone model with the simplicity of bandpass filtering detection. The proposed controller transitions from bandpass detection to the friction cone states once grasp stability is achieved. This strategy aims to balance autonomy between the user and the prosthesis, reducing user muscle effort and cognitive load in unexpected events. Healthy individuals naturally grasp objects with minimal conscious effort, as fine grasp geometry and responses to perturbations are handled with reflex actions. By transferring certain reflex behaviors to the prosthesis control during grasp instabilities, we aim to enhance interaction and reduce cognitive load without compromising user volition. Therefore, continuous monitoring of EMG inputs should ensure that if user’s intention is detected and the low-level controller suspends operation, granting full control to the user for adjusting the grasp. Moreover, while we advocate for a simple on/off EMG direct control approach here, there is room for exploring more advanced algorithms that do not compromise the functionality offered by the slip control modules.

Custom 3-axis force sensors were placed in the fingertips and palm of a robotic hand to validate the proposed approach in comparison with the other three state-of-the-art methods. The experimental validation consists of performing the grasping of an object with a robotic hand and inducing slip using a robotic arm. We tested various objects and different initial closure levels for a total of 260 trials. Results demonstrate a more efficient slip counteraction for the proposed approach than for a traditional friction cone controller, and a lower force applied than a traditional bandpass controller. Preliminary experiments in humans confirm the proposed controller behavior resemblance to human physiology, with successful completion of object lifting.

Future work will focus on improving the control algorithm with the introduction of a second threshold to distinguish slip perturbations from movement artifacts. Additionally, we plan to enhance the experimental setup by directly integrating tactile sensors into the robotic hand design. Future endeavors will involve individuals with limb loss to gain essential insights into the acceptance and effectiveness of these reflex control techniques. The proposed method can be extended to prostheses with individualized finger actuators, allowing for localized grasp corrections based on individual finger forces. Finally, providing selected haptic feedback stimuli to the user, and conveying information about detected forces and slip events, could improve the user awareness and sense of ownership over the prosthesis.

## Data Availability

The authors confirm that the data supporting the findings of this study are available within the article.
